# Immune Phenotype and Functionality of *Mtb*-Specific T-Cells in HIV/TB Co-Infected Patients on Antiretroviral Treatment

**DOI:** 10.3390/pathogens9030180

**Published:** 2020-03-02

**Authors:** Lucy Mupfumi, Cheleka A.M. Mpande, Tim Reid, Sikhulile Moyo, Sanghyuk S. Shin, Nicola Zetola, Tuelo Mogashoa, Rosemary M. Musonda, Ishmael Kasvosve, Thomas J. Scriba, Elisa Nemes, Simani Gaseitsiwe

**Affiliations:** 1 Department of Medical Laboratory Sciences, Faculty of Health Sciences, University of Botswana, Gaborone, Botswana; tuelomogashoa@me.com (T.M.); kasvosvei@ub.ac.bw (I.K.); 2 Botswana Harvard AIDS Institute Partnership, Gaborone 320, Botswana; sikhulilemoyo@gmail.com (S.M.); rmusonda@gmail.com (R.M.M.); 3 South African Tuberculosis Vaccine Initiative, Institute of Infectious Disease and Molecular Medicine, Division of Immunology, Department of Pathology, University of Cape Town, Cape Town 7935, South Africatim.reid@uct.ac.za (T.R.); thomas.scriba@uct.ac.za (T.J.S.); elisa.nemes@uct.ac.za (E.N.); 4 Department of Immunology and Infectious Diseases, Harvard T.H. Chan School of Public Health, Boston, MA 02138, USA; 5 Sue & Bill Gross School of Nursing, University of California Irvine, Irvine, CA 92697, USA; ssshin2@uci.edu; 6 Infectious Diseases Division, University of Pennsylvania, Philadelphia, PA 19104, USA; nzetola@gmail.com; 7 Botswana-Upenn Partnership, Gaborone, Botswana

**Keywords:** immune activation, HLA-DR, CD38, treatment response

## Abstract

The performance of host blood-based biomarkers for tuberculosis (TB) in HIV-infected patients on antiretroviral therapy (ART) has not been fully assessed. We evaluated the immune phenotype and functionality of antigen-specific T-cell responses in HIV positive (+) participants with TB (n = 12) compared to HIV negative (−) participants with either TB (n = 9) or latent TB infection (LTBI) (n = 9). We show that the cytokine profile of Mtb-specific CD4+ T-cells in participants with TB, regardless of HIV status, was predominantly single IFN-γ or dual IFN-γ/ TNFα. Whilst ESAT-6/CFP-10 responding T-cells were predominantly of an effector memory (CD27−CD45RA−CCR7−) profile, HIV-specific T-cells were mainly of a central (CD27+CD45RA−CCR7+) and transitional memory (CD27+CD45RA+/−CCR7−) phenotype on both CD4+ and CD8+ T-cells. Using receiving operating characteristic (ROC) curve analysis, co-expression of CD38 and HLA-DR on ESAT-6/CFP-10 responding total cytokine-producing CD4+ T-cells had a high sensitivity for discriminating HIV+TB (100%, 95% CI 70–100) and HIV−TB (100%, 95% CI 70–100) from latent TB with high specificity (100%, 95% CI 68–100 for HIV−TB) at a cut-off value of 5% and 13%, respectively. TB treatment reduced the proportion of *Mtb*-specific total cytokine+CD38+HLA-DR+ CD4+ T-cells only in HIV−TB (*p* = 0.001). Our results suggest that co-expression of CD38 and HLA-DR on *Mtb*-specific CD4+ T-cells could serve as a TB diagnosis tool regardless of HIV status.

## 1. Introduction

Tuberculosis (TB) results in an estimated 1.5 million deaths each year, making it the number one cause of death by a single infectious organism [1]. The burden of TB is disproportionately higher in Africa, where the co-infection rate with HIV is also the highest; more than 50% of the TB cases are HIV positive (+) [1]. The risk of active TB increases up to 30 times with HIV infection, with an annual risk of 15% compared to a 10% lifetime risk in HIV-uninfected participants [2]. Although antiretroviral therapy (ART) significantly reduces this risk [3], the incidence of TB while on ART remains higher than that in HIV-uninfected individuals [4,5]. HIV also specifically targets *Mycobacterium tuberculosis* (*Mtb*)-specific CD4 T-cells, resulting in impaired immune responses to TB in co-infected individuals [6,7]. 

Untreated HIV infection induces changes in the activation, memory, and functional profile of both CD4+ and CD8+ T-cells, with a decrease in naïve cells, and accumulation of highly differentiated cells [8]. It is hypothesized that this depletion of memory cells sustains the incidence of opportunistic infections in participants with advanced HIV. Uncontrolled replication of *Mtb* is associated with loss of CD27 expression [9], probably related to increased cellular homing to sites of disease. Recently, a ratio based on CD27 median fluorescent intensity (MFI) on CD4+ T-cells compared to that on IFN-γ+CD4+ T-cells has been shown to differentiate active disease from infection [10]. However, whether this approach has good diagnostic potential in HIV+TB remains to be determined.

Studies have also shown that activated effector *Mtb*-specific CD4+ T-cells characterized by the phenotype CD38+HLA-DR+ effectively distinguish latent TB infection (LTBI) from active TB cases [11,12,13] Furthermore, the frequency of *Mtb*-specific CD4+ T-cells expressing CD38 and HLA-DR declined rapidly within the first month of anti-tuberculosis treatment (ATT) [11,14]. CD38 expression on bulk CD8+ T-cells has long been recognized as a marker for disease progression in HIV-infected participants [15]. In addition, activated HLA-DR+ bulk and antigen-specific CD4+ T-cells have been shown to be a risk factor for TB progression in BCG-vaccinated infants [16] and to effectively discriminate latent from active TB in adults, respectively [13,14]. However, these studies were conducted predominantly in HIV− or HIV+ ART naïve individuals. It remains unclear what the impact of combined ART and ATT on the activation profile of *Mtb*-specific T-cells is.

ART seems to partially correct the HIV-induced T-cell defects and the activation profile of bulk T-cells from patients on ART is higher than that in HIV-uninfected individuals [17]. In a study of HIV-infected women who were followed up for a year post ART initiation, viral suppression and CD4+ T-cell increase did not lead to a concomitant decline in early (CD27+CD45RO+) and terminally differentiated (CD27−CD45RO+) memory CD4+ T-cells [17]. Since the memory phenotype determines the extent to which pathogen-specific responses are restored while on ART, persistent defects in the memory subsets of *Mtb*-specific CD4+ T-cells could account for the risk of active TB while on ART [18]. Therefore, it remains unclear how long-term ART affects the normalization of *Mtb*-specific T-cell memory phenotypes. Thus, in order to understand the dynamics of *Mtb*-specific T-cells during combined ART and ATT, we assessed the functional, activation, and differentiation profile of *Mtb*-specific T-cells in HIV/TB co-infected participants on ART compared to HIV− active TB. We also assessed whether the effects of ATT on immune responses were restricted to *Mtb*-specific T-cells by adding a Gag stimulation condition as a control for HIV-specific responses.

## 2. Results

### 2.1. Cohort Characteristics

**Figure 1:**
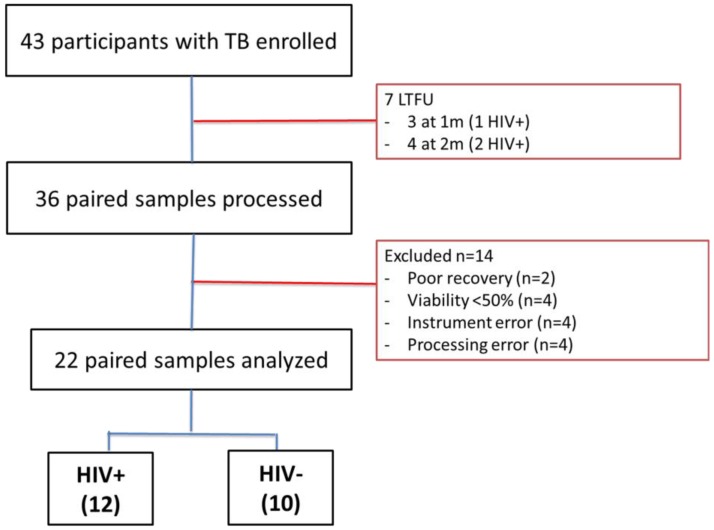
Study flow diagram. Samples were excluded for the following reasons: no follow-up sample available (n = 7), poor sample viability (n = 6), or technical errors (n = 8).

We restricted analysis to participants who had samples at TB diagnosis and two months post ATT to allow the determination of changes in the activation and functional phenotype of *Mtb*-specific T-cells due to ATT. We enrolled 43 participants with pulmonary TB and excluded samples from 14 participants due to poor specimen viability (n = 6) and technical (instrument/processing) errors (n = 8, Figure 1). A total of 30 samples were analyzed: 12 HIV positive (HIV+TB), 9 HIV negative (HIV−TB) and 9 HIV-LTBI. HIV+TB participants had a median age of 39 years (Q1, Q3: 33, 48) and had been on ART for a median of 1.3 years (Q1, Q3 0.2, 6, Table 1). All participants reported a cough at baseline and had culture negative results after two months of ATT.

### 2.2. Single IFN-γ and Dual IFN-γ/TNFα Response Characterizes Active Tuberculosis Regardless of HIV Status

**Figure 2:**
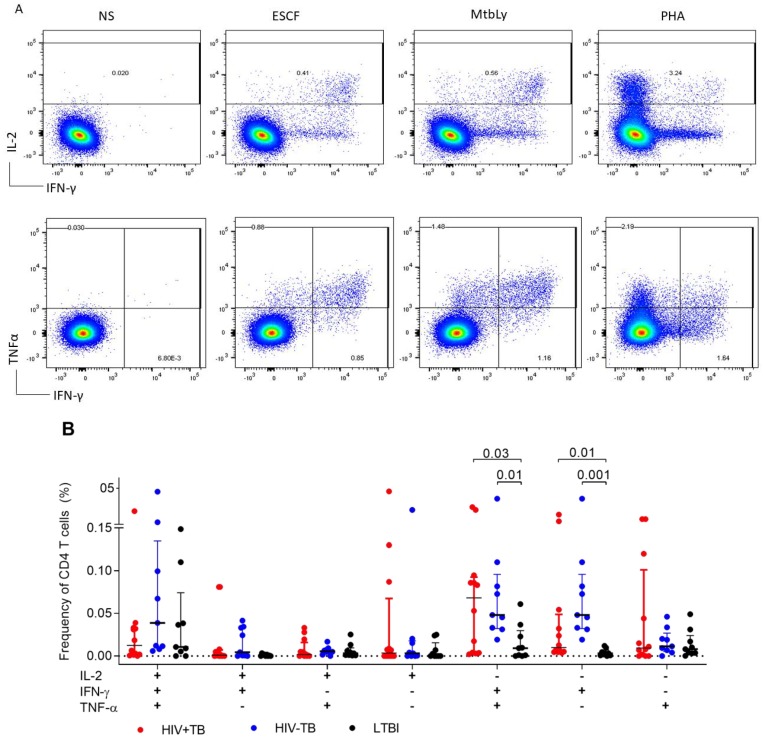
Comparison of the polyfunctional capacity of ESAT-6/CFP-10 CD4+ T-cells between HIV+TB, HIV−TB, and LTBI. (**A**) Representative plot of the expression of IL-2, *IFN-γ*, and TNF*α* in an HIV+TB participant. NS = unstimulated, ESCF = ESAT-6/CFP-10, MtbLy = Mtb-Lysate, PHA = Phytohemaglutinin. (**B**) Frequencies of CD4+ T-cells producing any of the possible combinations of IL-2, *IFN-γ*, and TNF*α* in response to ESAT-6/CFP-10 stimulation. Horizontal bars show the median and first and third quartile (Q1, Q3). Only statistically significant differences by the Kruskal–Wallis test with Dunn’s correction for multiple comparisons are shown on the graph.

We compared the magnitude of cytokine responses between TB and LTBI participants. Representative dot plots of the cytokine response to the four stimulation conditions are shown in the top panel of Figure 2A. At the time of TB diagnosis, IL-2−IFN-γ+TNFα+CD4+ T-cell responses to ESAT-6/CFP-10 stimulation were significantly higher in TB compared to LTBI but did not differ by HIV status (Figure 2B). 

**Figure 3:**
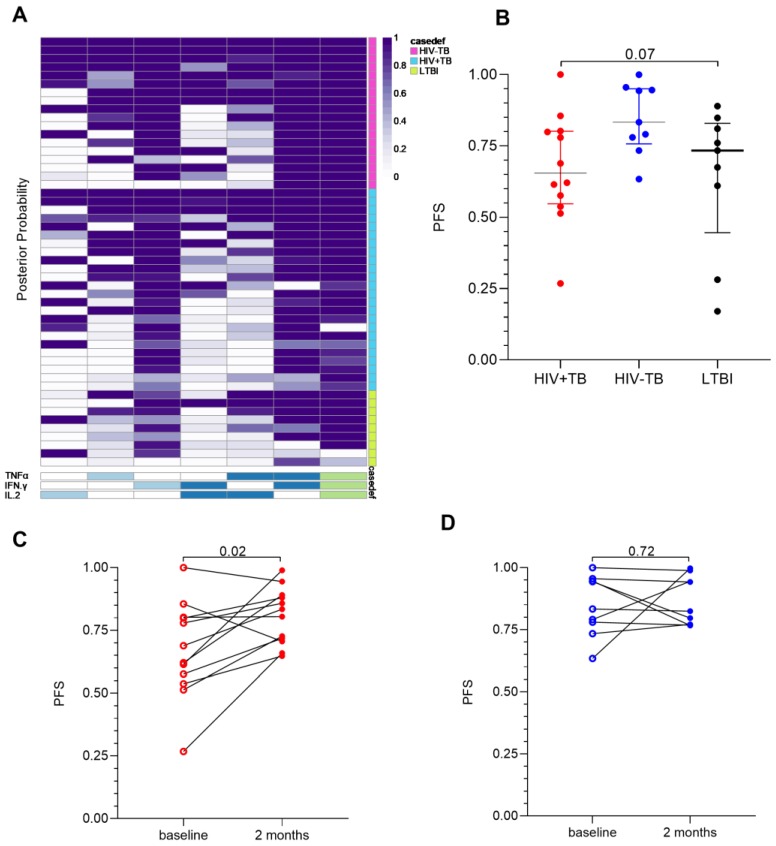
Polyfunctionality analysis of ESAT-6/CFP-10 specific CD4+ T-cell responses (**A**) Heatmap of combinatorial polyfunctionality analysis of antigen-specific T-cells (COMPASS) posterior probabilities of the distribution of responses in ESAT-6/CFP-10 stimulated CD4+ T-cells. Columns correspond to the different cell subsets modeled using the COMPASS package in R [19], color coded by the cytokines they express (white = “off”, shaded = “on”, grouped by color = “degree of polyfunctionality”) ordered by degree of functionality from one function on the left to all three functions on the right (shaded from light blue to green on the bottom). Rows correspond to participants; one on each line, the color of each cell represents the probability (range 0–1) that the cell subset exhibits an antigen-specific response. Each cell of the heatmap shows the probability that a given cell-subset (column) has an antigen-specific response in the corresponding participant (the groups are coded on the right of the heatmap; pink = HIV+TB, blue = HIV−TB, and green = LTBI). Plots of polyfunctional scores (PFS) stratified by patient group (**B**) and timepoint for HIV+TB (**C**) and HIV−TB (**D**)**.** Red dots represent HIV+TB, blue dots HIV−TB, and black dots LTBI. Differences between groups were compared using the Kruskal–Wallis test with Dunn’s correction for multiple comparisons are shown on the graph. We assessed differences between timepoints using the Wilcoxon signed-rank test.

In a similar manner, single IFN-γ+ responses were higher for both HIV+TB (*p* = 0.01) and HIV−TB (*p* = 0.001) compared to LTBI (Figure 2). There was no difference in the frequencies of polyfunctional (*p* = 0.12) or single IFN-γ responses between HIV+TB and HIV−TB (*p* = 0.33). This was also reflected in the combinatorial polyfunctionality analysis of antigen-specific T-cells (COMPASS) heatmap (Figure 3A), which showed similar probabilities for the polyfunctional and dual expression of IFN-γ/TNFα for both TB groups. We compared the polyfunctionality score (PFS) across groups, which summarizes the functional profile of each participant by calculating the posterior probabilities of antigen-specific responses [19]. There was no difference in the polyfunctional scores (PFS) by either TB or HIV status (*p* = 0.07, Figure 3B). Interestingly, when we analyzed Gag stimulated CD8+ T-cells, no polyfunctional responses were observed, instead, responses were characterized by single IFN-γ expression (Supplementary Figure S2A). We also found an increase in the PFS of ESAT-6/CFP-10-stimulated CD4+ T-cells at 2 months post ATT only in the HIV+TB (*p* = 0.02 compared to baseline PFS, Figure 3C), but not in HIV−TB (*p* = 0.72, Figure 3D). We did not observe any other differences in the cytokine profile between the baseline and two-month timepoint (results not shown). These data suggest that the frequency of polyfunctional *Mtb*-specific T-cells may not immediately reflect the reduction in mycobacterial burden due to TB treatment. 

**Figure 4:**
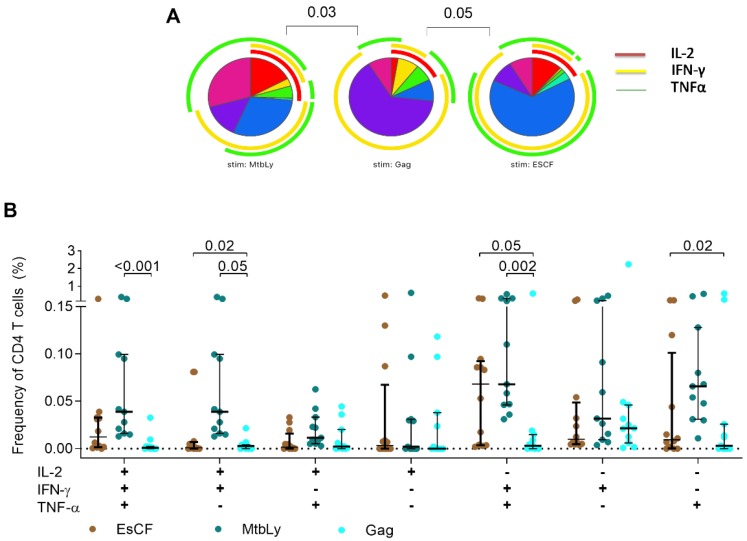
Functional analysis of HIV+TB. The functional profile of CD4+ T-cells specific for different antigens was assessed for CD4+ T-cells from HIV+TB patients at the time of TB diagnosis. (**A**) Each slice of the pie corresponds to a distinct combination of cytokines shown in (**B**) and defined by Boolean analysis in FlowJo. A key to the colors used for the arcs in the pie charts is shown on the top right. Pie charts were compared using the SPICE permutation test with *p* < 0.05 considered significant. (**B**) Frequency of cells producing any possible combination of IFN-γ, IL-2, or TNFα in response to the different stimulations. Horizontal bars represent median and interquartile range (IQR). Statistical comparisons were made using Kruskal–Wallis with Dunn’s test for multiple comparisons. MtbLy = Mtb-Lysate; ESCF = ESAT-6/CFP-10.

We also compared CD4 responses in the HIV+TB participants across stimulation conditions and observed that the relative proportions of polyfunctional and single TNFα response were highest on Mtb-Lysate-stimulated cells compared to Gag (*p* = 0.03; Figure 4A). In addition, dual IFN-γ/TNFα was higher on Mtb-Lysate (*p* = 0.002) and ESAT-6/CFP-10 (*p* = 0.05) compared to Gag-stimulated CD4+ T-cells (Figure 4B). This was also reflected on the COMPASS heatmap that showed similar PFS with no distinct cytokine patterns separating the groups analyzed (Supplementary Figure S2B). We further analyzed the functional profile of *Mtb*-specific CD4+ T-cells by comparing the MFI of TNFα on total cytokine+ cells. While the TNFα MFI on ESAT-6/CFP-10 stimulated CD4+ T-cells did not differ by HIV or TB status (*p* = 0.15, results not shown), we observed a decline at two months of TB treatment in HIV−TB (*p* = 0.02) but not HIV+TB (*p* = 0.27) (Supplementary Figure S3). 

### 2.3. Frequencies of CD38+HLA-DR+CD4+ T-cells Are Elevated in Active TB Participants Regardless of HIV Status and Decline after Two Months of TB Treatment

**Figure 5:**
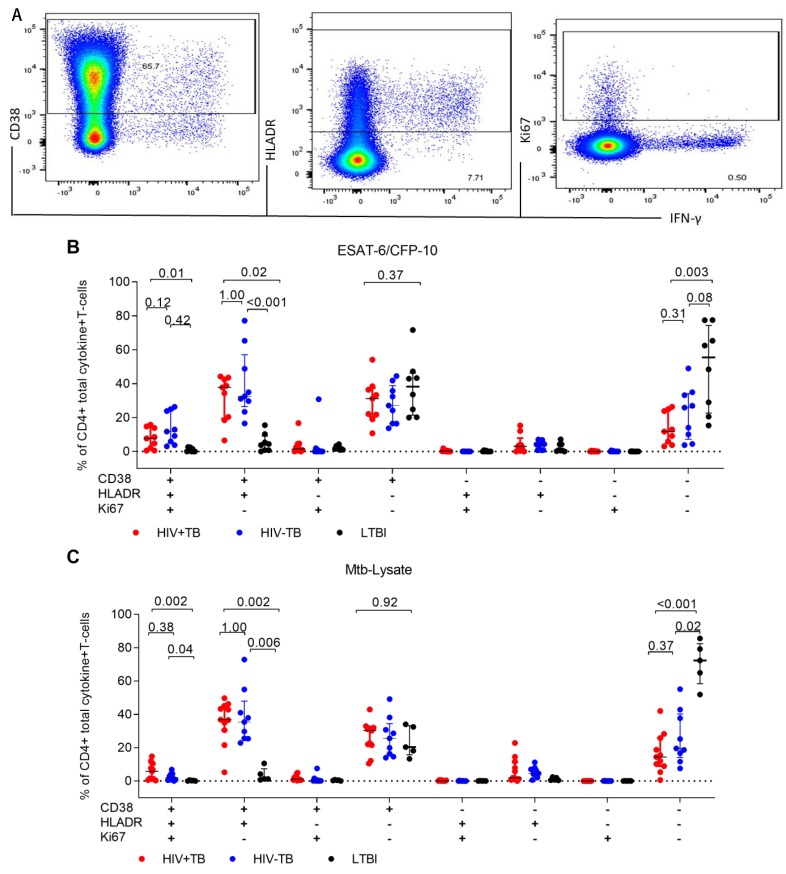
Comparison of the activation profiles of *Mtb*-specific CD4+ T-cells between HIV+TB, HIV−TB, and LTBI participants. (**A**) Representative dot plots of the activation profile on CD4+ T-cells of an HIV+TB patient in response to ESAT-6/CFP-10 stimulation. The proportion of each subset on ESAT-6/CFP-10 (**B**) and Mtb-Lysate (**C**) stimulated total cytokine+CD4+ T-cells. Statistical comparisons were made using the Kruskal–Wallis with Dunn’s test for multiple comparisons. We used Boolean gates to derive all possible combinations of the co-expressed markers.

We hypothesized that the activation profile of *Mtb*-specific CD4+ T-cells might differ by HIV status. We evaluated the expression of CD38, HLA-DR, and Ki67 alone or in combination on ESAT-6/CFP-10, Mtb-Lysate, and Gag-stimulated total cytokine+CD4+ T-cells. Representative dot plots of the expression of CD38, HLA-DR, and Ki67 on total cytokine CD4+ T-cells are shown in Figure 5A. All individuals tested had a positive CD4+T-cell response to Mtb-Lysate while 9/12 (75%) HIV+TB and 8/9 (89%) LTBI individuals had responses to ESAT-6/CFP10. We observed on ESAT-6/CFP-10-stimulated CD4+ T-cells that the triple expression of CD38+HLA-DR+Ki67+ was associated with active TB only in HIV+TB participants when compared to LTBI (*p* = 0.01), although there was no difference between HIV+TB and HIV−TB (*p* = 0.12; Figure 5B). On Mtb-Lysate stimulated cells, differences in the expression of CD38+HLA-DR+KI67+ CD4+ T-cells were observed between HIV+TB and LTBI (*p* = 0.002) and HIV−TB and LTBI (*p* = 0.04, Figure 5C).

**Figure 6:**
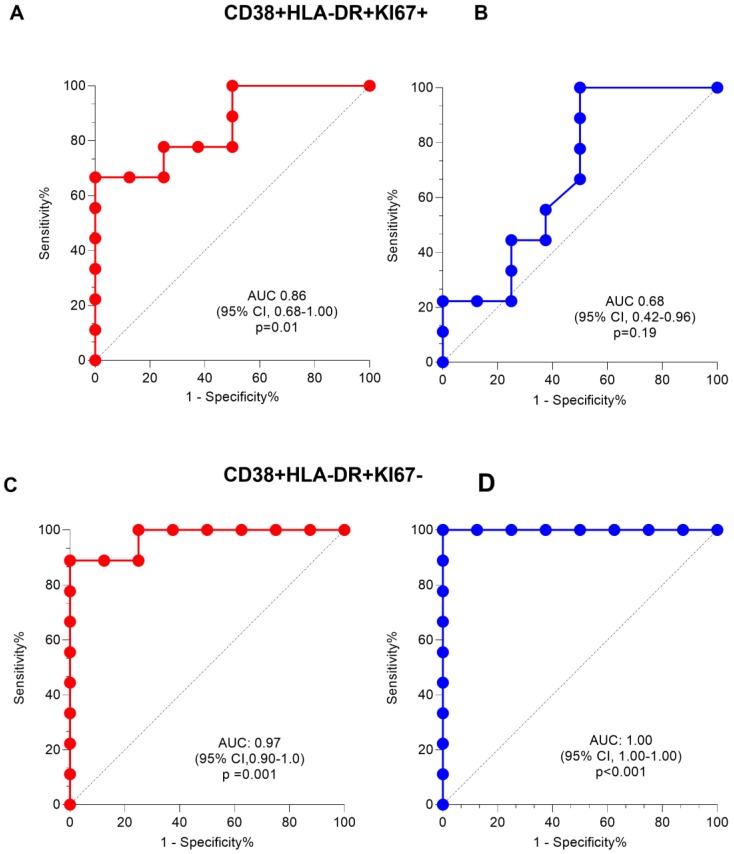
**Receiver operating characteristics** (ROC) curve analysis of the activation phenotype on total cytokine producing CD4+ T-cells for discriminating between TB and LTBI. Phenotypic analysis was only conducted in participants with a positive ESAT-6/CFP-10 response defined by a false discovery rate (FDR) < 0.05 using mixture models for single cell assays (MIMOSA) analysis: HIV+TB (n = 9); HIV−TB (n = 9); LTBI (n = 8). Comparison of the area under the curve for the phenotype CD38+HLA-DR+Ki67+ (top panel, **A**,**B**) and CD38+HLA-DR+Ki67- (bottom panel, **C**,**D**) in HIV+TB (red) and HIV−TB (blue). ROC curve analysis was done in Prism. The area under the curve (AUC) and p-values are shown. The dotted lines represent an AUC of 0.5.

In addition, the predictive ability of the triple activation profile in discriminating active from latent TB was high only in HIV+TB (AUC = 0.86, 95% CI 0.68–1.00, *p* = 0.01, Figure 6A), corresponding to a sensitivity of 78% (95% CI 45–96) and a specificity of 75% (95% CI 41–96) at a cut-off of 1.8%. The sensitivity in HIV−TB was low at the same cut-off (33% vs. 78%) with comparable specificity (75%) and an AUC = 0.68 (95% CI 0.42–0.96, *p* = 0.19, Figure 6B). Sensitivity improved with Mtb-Lysate stimulation to 100% (95% CI 74–100) for HIV+TB with similar specificity (80%, 95% CI 38–99) at a cut-off value of 0.30 (results not shown). In contrast, CD38+HLA-DR+Ki67− expression on ESAT-6/CFP-10-stimulated CD4+ T-cells had a high sensitivity for both HIV+TB (100%, 95% CI 70–100) and HIV−TB (100%, 95% CI 70–100) and modest specificity (63%, 95% CI 31–86 for HIV+TB and 88%, 95% CI 53–99 for HIV−TB) at a cut-off value of 5% and 13%, respectively. The corresponding ROC curves are shown in Figure 6C,D. On Gag-stimulated CD4+ T-cells of HIV+TB, the dominant activation profile was CD38+HLA-DR−Ki67−, similar to bulk CD4+ T-cells (results not shown) while CD8+ T-cells showed both CD38+HLA-DR+Ki67− and CD38+HLA-DR−Ki67− phenotypes (results not shown). When we evaluated the effect of TB treatment on the activation profile of ESAT-6/CFP-10-stimulated CD4+ T-cells, we observed significant declines in the proportion of CD38+HLA-DR+ CD4+ T-cells of HIV−TB (*p* = 0.001, Supplementary Figure S4A). In HIV+TB, only the CD38+HLA-DR+Ki67+ phenotype on ESAT-6/CFP-10 stimulated CD4+ T-cells declined at two months (*p* = 0.04, Supplementary Figure S4B). On Gag stimulated total cytokine+ CD8+ T-cells, we did not observe significant declines in the frequencies of cells expressing the CD38+HLA-DR+Ki67− (*p* = 0.69) or CD38+HLA-DR−Ki67− (*p* = 0.78) phenotypes (Supplementary Figure S4C).

### 2.4. A High CD27MFI Ratio Discriminates Active TB from Latent TB and Declines after Two Months of TB Treatment

**Figure 7:**
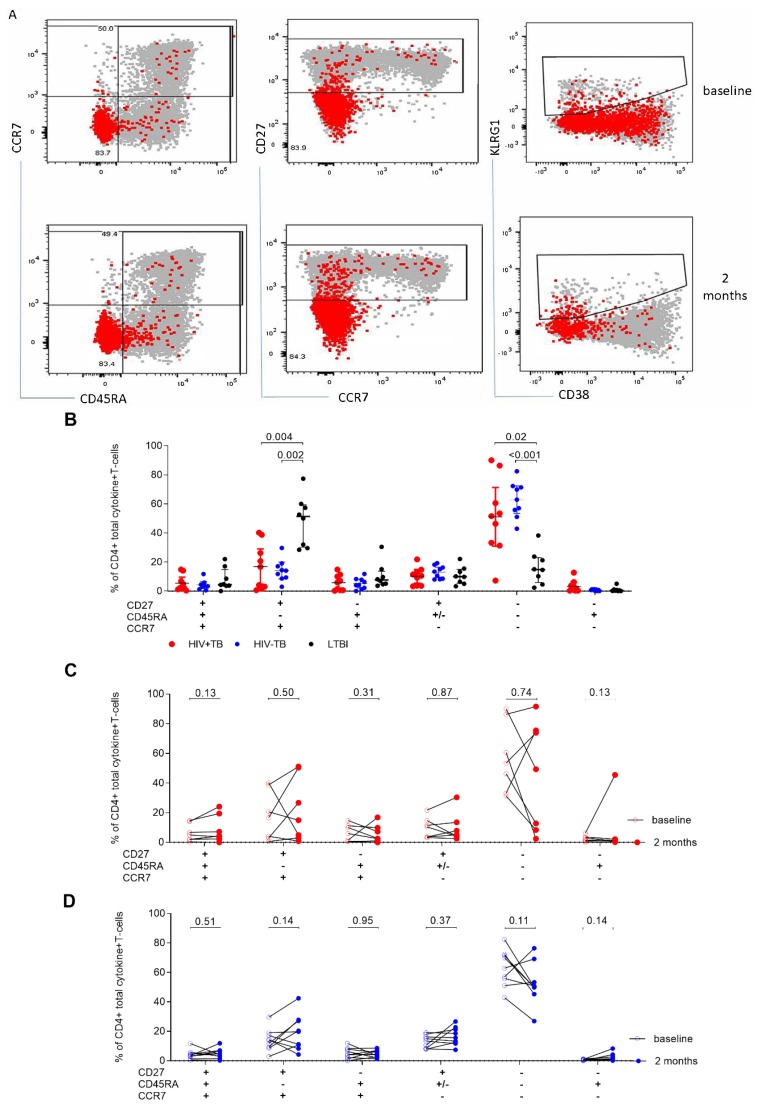
Comparison of the memory phenotype of ESAT-6/CFP-10-stimulated CD4+ T-cells between active TB and LTBI participants. (**A**) Representative plot of the total cytokine production in the memory compartment in response to ESAT-6/CFP-10 stimulation at baseline and two months (fu) timepoints. (**B**) Comparison of the memory phenotype of ESAT-6/CFP-10 stimulated total cytokine+CD4+cells between HIV+TB and HIV−TB. Changes in the memory phenotype between baseline and two months of TB treatment in HIV+TB (red, (**C**)) and HIV−TB (blue, (**D**)). Statistical comparisons were made using Kruskal–Wallis with Dunn’s test for multiple comparisons. Differences between time points were assessed using the Wilcoxon signed rank test.

To assess the effect of active disease on the memory phenotype of *Mtb*-specific CD4+ T-cells, we analyzed the Boolean combination of CD27, KLRG1, CD45RA, and CCR7 (Figure 7A shows representative dot plots). However, KLRG1 added no further information to the classification of memory subsets, therefore we used CD27, CD45RA, and CCR7 to define the memory phenotype. In agreement with previous reports, CD27 expression was downregulated on ESAT-6/CFP-10-stimulated CD4+ T-cells with an effector memory phenotype characterized by a high proportion of CD27−CD45RA−CCR7− within the total cytokine+ compartment (*p* < 0.001 for HIV−TB vs. LTBI and *p* = 0.02 for HIV+TB vs. LTBI, Figure 7B). In contrast, Gag-stimulated CD4+ T-cells were predominantly of a central memory phenotype (CD27+CD45RA−CCR7+; Supplementary Figure S5) with a dominant transitional memory phenotype (CD27+CD45RA+/−CCR7−; results not shown) observed on CD8+ T-cells. We did not observe statistically significant differences when we assessed the effect of TB treatment on the memory phenotype of ESAT-6/CFP-10-stimulated T-cells (Figure 7C,D).

**Figure 8:**
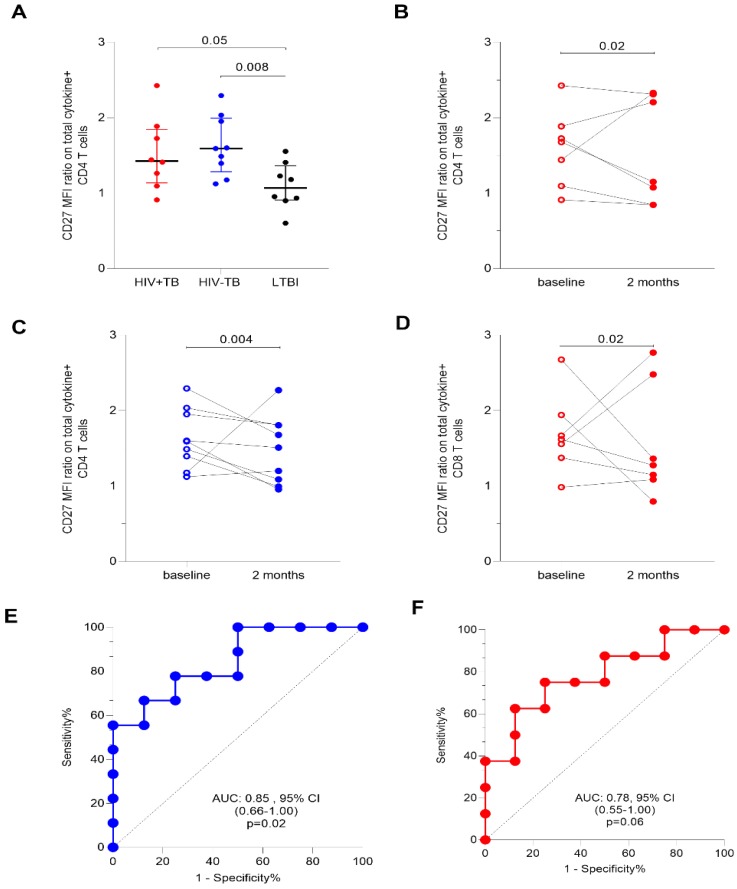
Sensitivity of the CD27MFI ratio on ESAT-6/CFP-10-specific CD4+ T-cells to detect active TB. (**A**) Comparison of the CD27MFI ratio between active TB and latent TB. CD27MFI ratio was calculated as the CD27MFI on bulk CD4 T-cells divided by CD27MFI on total cytokine+ CD4+ T-cells. Change in the CD27 MFI ratio of total cytokine+CD4+ T-cells between baseline and two months of TB treatment in (**B**) HIV+TB, (**C**) HIV−TB, and Gag-stimulated total cytokine+CD8+T-cells (**D**). Differences between time points were assessed using the Wilcoxon signed rank test. (**E**) and (**F**) show the receiver operating characteristics (ROC) curves of the CD27 MFI ratio in HIV−TB (blue) and HIV+TB (red), respectively, as assessed by the ROC curve analysis in Prism. The dotted lines represent an area under the curve (AUC) of 0.5.

We next evaluated CD27 expression as a ratio of the MFI of CD27 on CD4+ T-cells to that of CD27 on total cytokine+ CD4+ T-cells, using a modification of the calculation as suggested by Portevin et al. [10]. We observed that a high ratio was associated with active TB on ESAT-6/CFP-10 (*p* = 0.01 for HIV−TB vs. LTBI and *p* = 0.05 for HIV+TB vs. LTBI, Figure 8A), but not the Mtb-Lysate stimulated CD4+ T-cells (*p* = 0.10; results not shown). Significant declines in the ratio on ESAT-6/CFP-10-specific CD4+ T-cells were observed at two months of treatment in both HIV+TB (*p* = 0.02, Figure 8B) and HIV−TB (*p* = 0.004, Figure 8C). This decline was also observed on Gag stimulated CD8+T-cells of HIV+TB (*p* = 0.03, Figure 8D). We then assessed the diagnostic accuracy of using the CD27 MFI ratio on ESAT-6/CFP-10 stimulated CD4+ T-cells and found comparable sensitivity in HIV−TB (67%, 95% CI 35–88) and HIV+TB (63%, 95% CI 31–86) at a cut-off of 1.4. The corresponding ROC curves are shown in Figure 8E,F. Overall, our results suggest that an approach using the CD27MFI ratio on ESAT-6/CFP-10-specific total cytokine+ CD4+ T-cells could be used to discriminate between disease and infection in HIV-uninfected individuals and monitor treatment response during active TB, regardless of HIV status.

## 3. Discussion

Our results show that the activation and memory phenotype of *Mtb*-specific CD4+ T-cells can be used to discriminate between active TB and LTBI as well as to monitor declines in mycobacterial burden after two months of ATT. These findings extend the results previously reported in ART naïve participants [12,13,20] to individuals on ART. 

Conflicting reports on the assessment of the functional profile in HIV+TB have been published, reflecting the challenges of relying on cytokine profiles as biomarkers of TB. While some studies have shown that in active TB there is a depletion of single IFN-γ expression [21,22,23] accompanied by a dominant TNFα profile [24], others have reported that a polyfunctional profile is associated with HIV+TB cases [25]. We observed dominant single IFN-γ and dual functional IFN-γ+TNFα+ CD4+ T-cells in response to ESAT-6/CFP-10 and Mtb-Lysate stimulation consistent with reports from South African cohorts [12,13]. Whether these inconsistencies are due to differences in demographics of the study cohorts, study design, or flow cytometry technical differences is not very clear. More useful than the polyfunctional profile was the PFS that increased in response to ATT in HIV−TB, although there was no difference in scores between HIV+TB and HIV−TB. Therefore, using functional profiles as assessed by COMPASS may be a better measure of response to treatment. Stimulation with Mtb-Lysate also revealed a dominant single TNFα response that had not been evident on ESAT-6/CFP-10 CD4+ T-cells. In addition, all participants responded to Mtb-Lysate, which suggests that this antigen formulation may offer advantages for detecting responses in participants who are unresponsive to ESAT-6/CFP-10 [13]. This is because Mtb-Lysate detects mycobacterial responses induced by Mtb, BCG vaccination, and/or exposure to environmental mycobacteria, [13] which could be a drawback on the specificity of the responses obtained.

HIV is a major risk factor for TB, with deficiencies in *Mtb*-specific CD4+ T-cells possibly contributing to the risk of active TB prior to declines in peripheral blood CD4+ T-cells [26]. While ART-induced immune reconstitution is well described, the impact of ART on co-pathogen specific CD4+ T-cell responses is poorly understood [17,27]. For example, although ART induced declines in bulk CD4+ and CD8+ T-cells co-expressing CD38 and HLA-DR after a year of ART, the activation was still higher than on T-cells from HIV-uninfected individuals [18]. Since the activation level of T-cells is one of the factors influencing the extent of immune restoration [18], the use of blood-based markers may be an effective tool to assess the risk of active TB prior to ART initiation. In our cohort of participants who had been on ART for a median of one year, we found that the immune activation in HIV+TB, measured as a proportion of CD38+HLA-DR+totalcytokine+ ESAT-6/CFP-10-responding CD4+ T-cells, was comparable to HIV−TB but higher than LTBI at the time of TB diagnosis. In addition, significant declines in activation at two months post-ATT were observed only in HIV−TB. This could indicate that we needed a longer follow-up period and a larger sample size to observe a decline in response to TB treatment in the HIV+TB group.

Prior studies have shown that the activation profile of Mtb-specific CD4+ T-cells defined by CD38, HLA-DR, and Ki67 in combination [11,12,13] or alone [14] can distinguish latent from active TB with high sensitivity. Our results of a high sensitivity for the co-expression of CD38 and HLA-DR on *Mtb*-specific CD4+ T-cells as reflected by the ROC curve analysis agree with reports from Riou et al. [13] and Wilkinson et al. [12], in our analysis, the co-expression of CD38, HLA-DR, and Ki67 had poor sensitivity for TB diagnosis and the proportion of cells expressing this phenotype did not change after two months of TB treatment. The expression of Ki67 has also been shown to be a less robust marker for differentiating active disease from LTBI in a study of HIV infected participants who were not on ART [12].

The chronic antigen exposure that occurs in active TB disease drives the maturation of *Mtb*-specific CD4+ T-cells toward a late differentiated phenotype [28]. CD27 loss has been shown to differentiate active TB from LTBI [9,10,13,28] and associates with clinical disease severity and lung tissue damage in active TB [29]. Consistent with these reports, *Mtb*-specific CD4+ T-cells in response to ESAT-6/CFP-10 stimulation from active TB cases, regardless of HIV status, had a dominant effector memory phenotype compared to the central memory phenotype on LTBI. In contrast, Gag stimulated CD8+ T-cells had a dominant transitional memory phenotype that has been associated with HIV virologic control in participants not on ART and reduced immune activation [30].

Recently, a T-cell-activation marker-tuberculosis (TAM-TB) assay that measures the CD27 phenotype of IFN-γ producing cells was shown to have good performance characteristics in children [10]. We extended the analysis of the CD27 MFI ratio (measured as CD27 MFI on CD4+ T-cells divided by CD27 MFI on IFN-γ+CD4+ T-cells), suggested by Portevin et al. [10], to total cytokine+CD4+ T-cells, as recently reported [9]. We added new information regarding the CD27 MFI ratio in HIV+TB participants and showed that the ratio was highest on ESAT-6/CFP-10 responding total cytokine+ CD4+ T-cells in active TB participants regardless of HIV status. Previously, CD27 was analyzed as a % or MFI on IFN-γ+CD4+ T-cells of HIV+TB who were not on ART also showed good sensitivity in distinguishing active disease from infection [13]. In our analysis, TB treatment also caused declines in the CD27 MFI ratio in the HIV−TB participants. An increase in CD27 expression on *Mtb*-specific T-cells has also been shown to correlate with the conversion of sputum culture and lung repair measured as the reduction in lung tissue destruction on radiological examination at two months of ATT [29].

Our results should be interpreted in light of some limitations to the analyses. The sample size of our study groups was very small and was limited by studying participants with paired samples to detect early changes during ATT. This further reduced the sample sizes and may have possibly masked some differences and limited our ability to detect changes in certain phenotypes. Larger cohorts would be required to extend our findings. Whilst biomarkers for early treatment monitoring are important for identifying patients that are likely to respond well to shorter treatment regimens [31], the lack of an end of treatment timepoint and the fact that all patients had culture negative results at two months, limits our ability to comment on whether changes in the biomarkers are associated with favorable responses to ATT. Nonetheless, our results show that although TB treatment generally lasts six months, there are a number of patients that will culture convert by the end of the intensive phase of ATT. In addition, the inclusion of Gag stimulation allowed us to observe that these changes in T-cell activation appeared to be specific to the reduction of *Mtb* load during ATT because Gag-specific cells remained unaffected. However, the functional impact of the divergence in the memory phenotypes observed between Mtb and HIV-specific T-cells could not be determined due to the nature of the study design. Furthermore, we did not include HIV+ participants without TB as a control, which limits our ability to comment on the specificity of such responses.

Overall, our results show that CD38 and HLA-DR co-expression on *Mtb*-specific T-cells has good performance characteristics for active TB regardless of HIV status and has potential for early treatment monitoring in HIV+TB individuals on ART. The CD27 MFI ratio performed well on ESAT-6/CFP-10 responding total cytokine+ CD4+ T-cells in HIV−TB and needs further validation in large, well-powered cohorts to define its potential in monitoring early treatment responses in HIV+TB. 

## 4. Materials and Methods

### 4.1. Study Participants

Participants with a diagnosis of pulmonary TB were recruited from 10 primary health clinics in Gaborone, Botswana during the period December 2017 to August 2018. Participants were recruited on the day of TB diagnosis or within five days thereof, prior to anti-TB treatment (ATT). All participants had a confirmed TB diagnosis by either smear microscopy or X-pert MTB/RIF (Cepheid, Inc., Sunnyvale, CA, USA). Sputum samples were collected at the time of diagnosis and cultured using Lowenstein–Jensen media, and phenotypic drug susceptibility testing was conducted. We collected samples from 10 healthy participants with no symptoms or previous history of TB, with a positive Quantiferon Gold Plus test (QIAGEN, Hilden, Germany) to serve as a comparison group. Blood samples were collected from all participants in sodium heparin tubes for peripheral blood mononuclear cell (PBMC) isolation. Participants with TB had study visits at 1- and 2-months post ATT, with sputum samples for culture and blood for PBMC processing collected at each visit. Samples for CD4 and viral load testing were collected at the baseline and two months and processed on the FACSCalibur (BD Biosciences, San Jose, CA, USA) and Abbott m2000sp/rt (Abbott, Chicago, IL, USA) instruments, respectively, at the ISO-accredited Botswana Harvard HIV Reference Laboratory (BHHRL).

### 4.2. Laboratory Methods

#### 4.2.1. Cell Activation

PBMCs were isolated using the Ficoll–Hypaque density gradient method (GE Healthcare, Chicago, IL, USA) and stored in liquid nitrogen before shipping to the South African Tuberculosis Vaccine Initiative (SATVI) for processing. Cryopreserved cells were thawed and rested for two hours in Roswell Park Memorial Institute RPMI 1640 media containing 10% heat-inactivated fecal bovine serum (FBS) prior to antigen stimulation. We stimulated cells with a pool of early secretory antigen (ESAT-6) and culture filtrate protein (CFP-10, peptide pool referred to as ESCF) consisting of 17 and 16 peptides, respectively, overlapping by 10 amino acid sequences (1 µg/mL, GenScript, Piscataway, NJ, USA); *Mtb* cell lysate (MtbLy) (H_37_Rv; 10 µg/mL, BEI Resources, Manassas, VA, USA), and HIV-1C Gag 15 mers overlapping by 10 amino acids (2 µg/mL, BEI Resources, Manassas, VA, USA). Phytohemagglutinin antigen (PHA; 2 µg/mL) was used as a positive control. A negative control without stimulation (NS) was also included. Stimulations were performed for two hours in a 37 °C CO_2_ incubator, after which Brefeldin A (5 µg/mL, Sigma-Aldrich, St. Louis, MO, USA) was added and cells were further stimulated for four hours. All stimulations were conducted in the presence of the co-stimulatory antibodies, anti-CD28 and anti-CD49d (1 µg/mL, BD Biosciences, San Jose, CA, USA) for a total of six hours. 

#### 4.2.2. Cell Staining

After stimulations, cells were washed, stained with phycoerythrin (PE)-conjugated chemokine receptor antibody CCR7 (BD Biosciences, San Jose, CA, USA), and subsequently surface stained with the following antibodies: Brilliant violet (BV) 650-conjugated CD3 (BD Biosciences, San Jose, CA, USA); CD4-BV785 (BD); CD45RA-BV570 (BioLegend, San Diego, CA, USA); CD27–BV510 (BD Biosciences, San Jose, CA, USA); Fluorescein Isothiocynate (FITC)-conjugated HLA-DR (BioLegend, San Diego, CA, USA); Phycoerythrin (PECy5)-conjugated CD38 (BD Biosciences, San Jose, CA, USA); Peridinin-chlorophyll-protein complex (PerCP_eFluor710)-conjugated Killer Lectin-like receptor G (KLRG1, Invitrogen, Carlsbad, CA, USA); and LIVE-DEAD-Far Infrared (IR, Invitrogen, Shenzhen, China). The antibody panel including clone and catalog number in accordance with the MIFlowCyt guidelines [32] is shown in Supplementary Table S1. Cells were then fixed and permeabilized using Cytofix/Cytoperm buffer (BD Biosciences, San Jose, CA, USA) and stained with the antibodies Allophycocyanin (APC)-conjugated Interleukin (IL)-2 (BD Biosciences, San Jose, CA, USA); interferon gamma (IFN-γ)-AlexaFluor700 (BD Biosciences, San Jose, CA, USA); tumor necrosis factor (TNF)-α -PeCy7 (BioLegend, San Diego, CA, USA); and Ki67-BV421 (BD Biosciences, San Jose, CA, USA). Cells were fixed in 1% paraformaldehyde and acquired on a BD LSRII flow cytometer within one hour of fixation at the South African Tuberculosis Vaccine Initiative (SATVI). FACS data were analyzed in FlowJo (BD Biosciences, San Jose, CA, USA) and Boolean combination gating was used to calculate frequencies corresponding to eight and sixteen different combinations of cytokines and memory markers, respectively. The gating strategy is shown in Supplementary Figure S1.

### 4.3. Statistical Analysis

In order to compare the functional, activation, and memory profile of *Mtb*-specific CD4+ T-cells between antigen conditions or patient groups, we used the Wilcoxon rank sum, and Kruskal–Wallis with Dunn’s tests for multiple comparisons as appropriate. Cytokine combinations were assessed using SPICE (simplified presentation of incredibly complex evaluations) software and data are reported after background subtraction [33]. Differences in the functional, activation, and memory profile of *Mtb*-specific CD4+ T-cells between baseline and two months were analyzed using the Wilcoxon signed rank test. Receiver operating characteristics (ROC) curve analysis was used for single marker analysis. COMPASS (combinatorial polyfunctionality analysis of antigen-specific T-cells) and MIMOSA (mixture models for single cell assays) analysis was conducted in R (v1.1.463, The R Foundation for Statistical Computing, Vienna, Austria) using the packages as described previously [19]. To reliably measure the expression of phenotypic markers on antigen specific cells, we first determined whether the immune response to antigen stimulation was significantly higher when compared to non-specific cytokine production detectable in unstimulated samples. To do this, we applied MIMOSA analysis and defined antigen responders as participants with a false discovery rate (FDR) < 0.05 from this analysis. COMPASS utilizes a Bayesian hierarchical mixture model to identify antigen-specific changes simultaneously across all possible T-cell subsets [19]. Such an approach has a higher sensitivity and specificity for detecting true responses over alternative approaches such as basic log fold change [19]. A Markov Chain Monte Carlo algorithm computes posterior probabilities resulting in two scores; functionality and polyfunctionality that can be correlated with any outcome of interest. We analyzed data using STATA (v14.2; StataCorp, College Station, TX, USA) and GraphPad Prism (v8.1; GraphPad Software, La Jolla, CA, USA). A *p*-value < 0.05 was considered statistically significant and we adjusted for multiple comparisons where needed. 

### 4.4. Ethical Considerations

This work received ethical approval from The University of Botswana IRB and the Human Research Development Committee (IRB of the Botswana Ministry of Health and Wellness, No: HPDME 13/18/1). All participants provided written informed consent.

## Figures and Tables

**Table 1:** Baseline characteristics of the study participants.

Characteristic	HIV+TB (n = 12)	HIV−TB (n = 9)	LTBI (n = 9)
Age, median years (Q1, Q3)	39 (33, 48)	28 (25, 40)	31 (28, 36)
Female (%)	7 (58)	2 (22)	4 (44)
Previous TB diagnosis	2 (17)	0	0
Cough duration (%)			N/A
<1 m	8 (67)	0	
1–2	3 (25)	4 (44)	
2–3	1 (8)	3 (33)	
>3 m	0	2 (22)	
CD4 T-cell count, median (Q1, Q3)	288 (172, 357)	N/A	N/A
CD4:CD8 ratio, median (Q1, Q3)	0.77 (0.4, 1.12)	N/A	N/A
Viral load, copies/mL ^1^ median (Q1, Q3)	317,212 (76, 327,725)	N/A	N/A
ART duration, median years (Q1, Q3)	1.3 (0.2, 6)	N/A	N/A

^1^ Viral load results for three participants with detectable viral load at baseline; one had been on ART for 1.9 years and two were not on ART at the time, but were initiated on Dolutegravir (DTG)-based ART two weeks after ATT. By the two-month study visit, all patients had viral load results below 40 copies/mL.

## Supplementary Materials

The following are available online at https://www.mdpi.com/2076-0817/9/3/180/s1, Figure S1: Gating Strategy, Figure S2: COMPASS analysis of Gag and Mtb-Lysate-stimulated CD8 and CD4 T-cells, Figure S3: Comparison of TNFα expression within total cytokine+CD4 T-cells, Figure S4: Comparison of activation profile of Gag-stimulated total cytokine+CD8+T-cells, Figure S5: Comparison of memory phenotypes between Mtb and HIV specific total cytokine+CD4+T-cells, Table S1: Antibody Panel.
