# Supplementary material for: Immune Phenotype and Functionality of Mtb-Specific T-Cells in HIV/TB Co-Infected Patients on Antiretroviral Treatment
Source: Pathogens. 2020 Mar 2;9(3):180. doi: 10.3390/pathogens9030180 (PMC7157681; doi:10.3390/pathogens9030180)
Supplement: Supplementary file 1 [file pathogens-09-00180-s001.pdf]

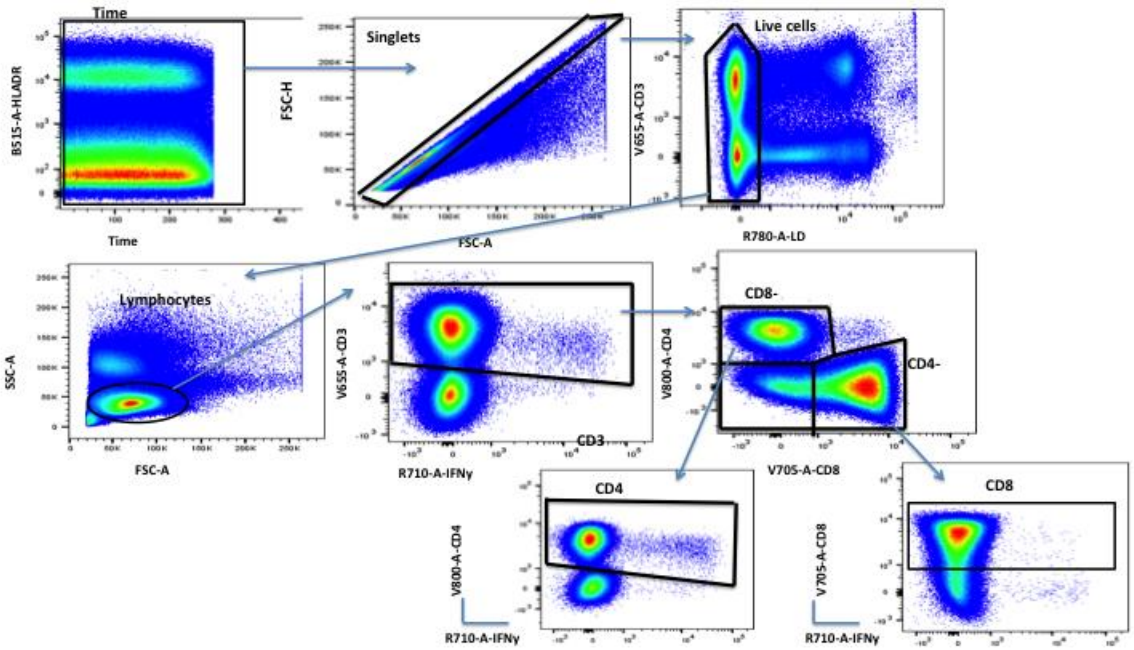

Fig S1: Gating strategy. After excluding dead cells and aggregates, we gated for CD4+ and CD8+ T-cells as shown.

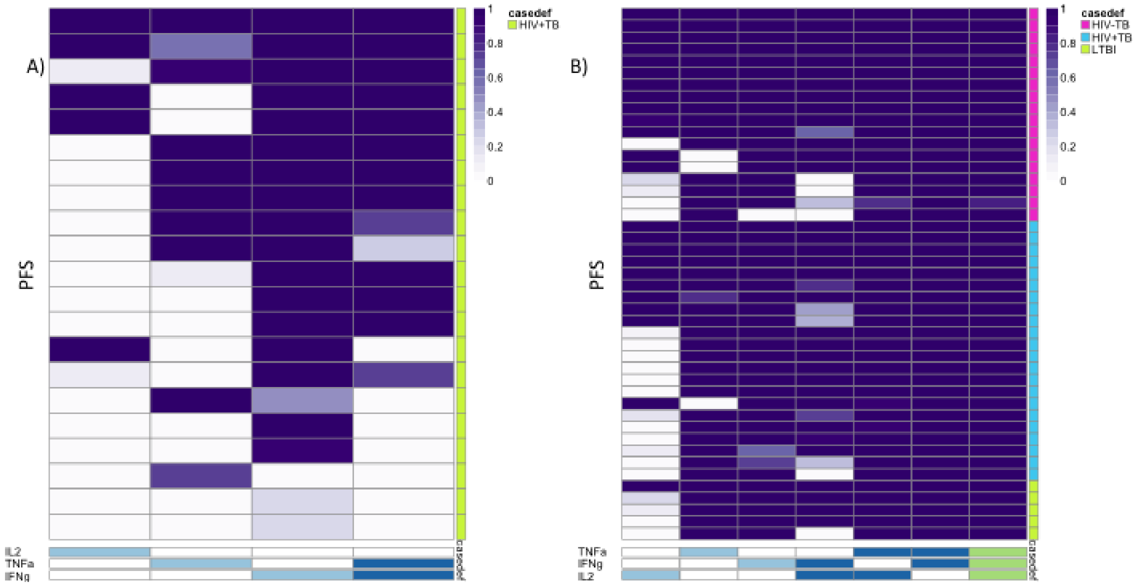

Fig S2: COMPASS analysis. Heatmap of COMPASS posterior probabilities of distribution of responses in A) Gag and B) Mtb-Lysate stimulated CD8+ and CD4+ T-cells respectively. Columns correspond to the different cell subsets modeled by the COMPASS package in R, color-coded by the cytokines they express (white="off", shaded="on", grouped by color="degree of polyfunctionality"). Subsets with maximum posterior probabilities <0.005 were removed from the Gag heatmap (a). Rows correspond to patients, one on each line. PFS=polyfunctional score.

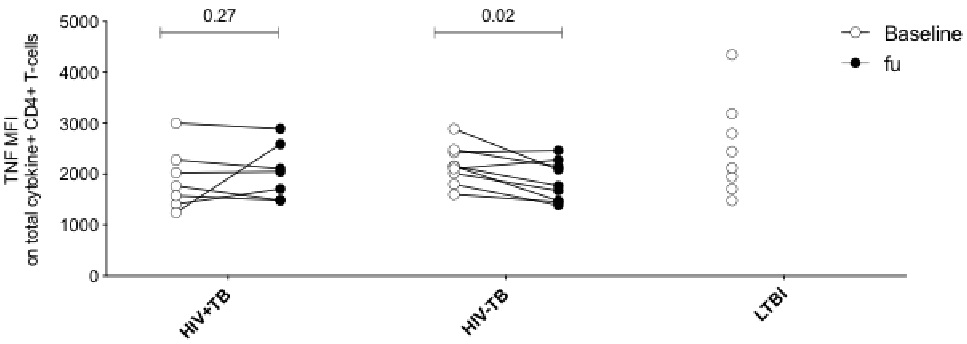

Fig S3: Comparison of TNFα expression levels within total cytokine+CD4+T-cells. Analysis was restricted to patients with responses at baseline and 2 months post TB-treatment as defined by an FDR<0.05 on Mimosa analysis [HIV+TB (n=7); HIV-TB (n=9); LTBI (n=8)]. Statistical comparisons were made using the Wilcoxon signed rank test.

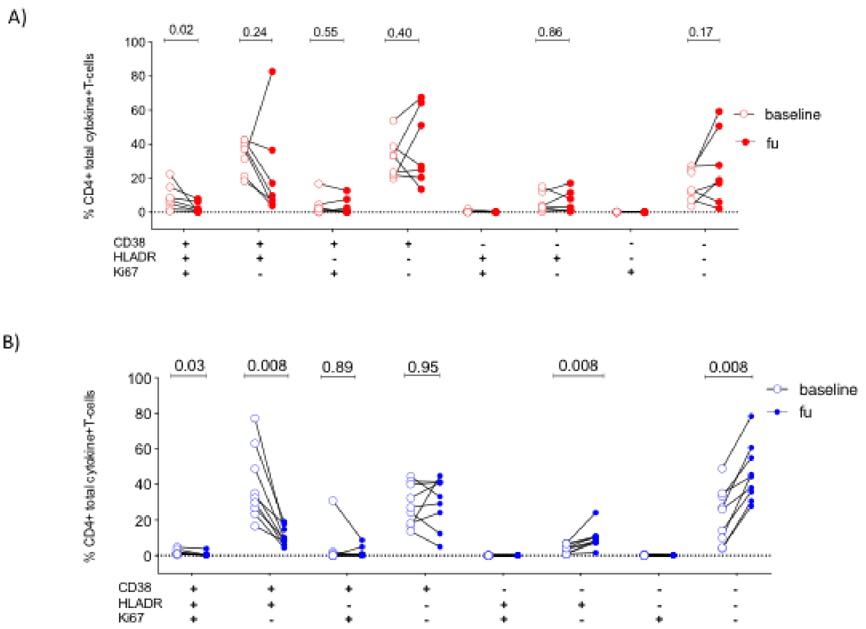

Fig S4: Comparison of activation profile on Gag-stimulated total cytokine+CD8+T-cells. Change in the activation profile of Esat-6/CFP-10 stimulated CD4+ T-cells at 2 months post TB treatment in HIV-aTB (blue, (a)) and HIV+aTB (red, (b)). Analysis was restricted to patients with responses at baseline and 2 months post TB-treatment as defined by an FDR<0.05 on Mimosa analysis. Statistical comparisons were made using Kruskall-Wallis with Dunn's test for multiple comparisons.

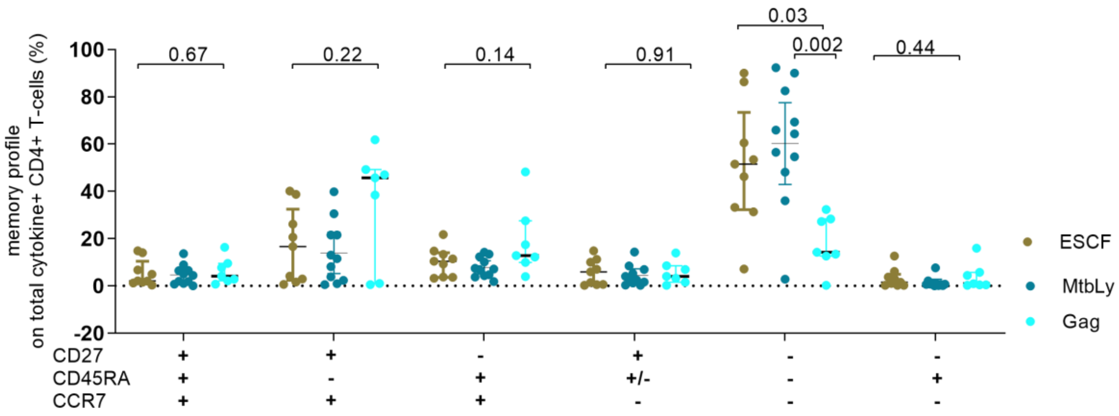

Fig S5: Comparison of memory phenotypes between *Mtb* and HIV specific total cytokine+CD4+T-cells. Differences between ESAT-6/CFP-10, Mtb-Lysate and Gag stimulated CD4+ T-cells at TB diagnosis in HIV+aTB . ESCF=ESAT-6/CFP-10, MtbLy=Mtb-Lysate. Analysis was restricted to patients with responses at baseline as defined by an FDR<0.05 on MIMOSA analysis. Statistical comparisons were made using Kruskall-Wallis with Dunn's test for multiple comparisons.

Table S1. Antibody Panel.

Characteristic	Marker	Fluorochrome	Clone	Cat #	Supplier
Cell Viability	Live/Dead	Far IR (APC-H7)	N/A	L10119	Invitrogen
T-cell Lineage	CD3	BV650	UCHT1	563852	BD
	CD4	BV785	OKT4	317442	BioLegend
	CD8	BV711	RPA-T8	301044	BioLegend
Intracellular Cytokines	IFN-◎	Alexa Fluor 700	B27	557995	BD
	IL-2	APC	Rat	554567	BD
	TNF-◎	PE-Cy7	Mab11	25734982	Invitrogen
T-cell Memory	CD45RA	BV570	HI100	304132	BioLegend
	CCR7	PE	150503	560765	BD
	CD27	BV510	L128	563092	BD
	KLRG-1	PercP-eFlour710	13F12F2	46948842	Invitrogen
T-cell Activation	HLA-DR	FITC	L243	307604	BioLegend
	CD38	PE-CY5	HIT2	555461	BD
	Ki-67	BV421	B56	562899	BD
